# Dislocation of Occipital Bone: Letter from Dr. W. H. Lancaster, Coleman, Texas

**Published:** 1886-11

**Authors:** W. H. Lancaster

**Affiliations:** Coleman, Texas


### DISLOCATION OF OCCIPITAL BONE FROM ATLAS.

*Editor Daniel's Texas Medical Journal:*

I herewith append you a report of a case, which will add one more to the very few reported cases to be found in medical literature. On September 26th, 1886, Dr. P. J. Powers was called urgently to visit, five miles in the country, Mr. Failing, who had been thrown from a falling horse, about 3 P. M., August 16th. The doctor arrived about 6 P. M., and found patient comatose, perfectly paralized except as to respiratory and cardiac function, respiration very stertorous, and at intervals intermitting. Right pupil quite dilated. Left contracted to pin point apperture, the pulse full, regular and 84 beats to a minute. Inferior maxilla dropped down; unable to swallow, temperature normal. No reaction whatever. The Doctor turned the patient in left semiprone position and examined the spine, as he thought, very carefully, but found nothing externally indicating any lesion of that part. No external evidence of injury was visible except dislocation of left shoulder, head of the humerus forward, downward, slightly, and inwards; subglenoid variety. This because of the perfect paralysis was easily reduced. There was no congestion of conjunctiva. Directions were given enjoining perfect rest with the exception of occasional stimulating pediluvia awaiting morning for some reaction. At 3 P. M., next day, patient was again seen by Dr. B. and myself. Condition of patient changed in the following respects: Temperature 104½, pulse 130, respiration 30 to a minute; quite an accumulation of sordes on teeth. On examining patient I found a dislocation of the occipital bone, from Atlas, and such as that when in the left semiprone position was almost replaced for the time, and difficult to discern, but in all other positions quite evident; and also such that if the head were placed in same position, the respirations could be interrupted, and indeed I thought that at one time by a certain position, an end had been reached; this, however, was relieved by a selected position. The abnormal position of the spinal column, or atlas was to right and possibly posteriorly;—occcipital bone of course to left. There was also slight spasms of left arm and hand, with extreme extension of both feet; no motion else, except those caused by respiration. Patient died at 6 P. M., 27th, twenty-seven hours after fall, without any increase of convulsions.

Respectfully,

W. H. LANCASTER, M. D.

Coleman, Texas, October 2nd, 1886.

